# Optimization of applied loads for assessing load-velocity relationship during back squat

**DOI:** 10.1371/journal.pone.0328772

**Published:** 2025-07-18

**Authors:** Zhaoqian Li, Litong Xiao, Xing Zhang, Changda Lu, Junbei Bai

**Affiliations:** 1 Department of Physical Education and Sport, Faculty of Sport Sciences, University of Granada, Spain; 2 Department of Military Training, Officers·College of Chinese People’s Armed Police Force, Chengdu, China; 3 School of Sport Science, Beijing Sport University, Beijing, China; 4 Competitive Sports No.4 Room, Beijing Research Institute of Sports Science, Beijing, China; University of Mississippi, UNITED STATES OF AMERICA

## Abstract

**Purpose:**

This study aimed to identify the lightest load to validate the load-velocity (L-V) relationship in the back squat using the modified multiple-point (a lower highest load compared to the standard method) and in-field two-point methods.

**Methods:**

Following the measurement of back squat one-repetition maximum (1RM), twenty college amateur athletes performed a multi-point incremental loads test (20%, 40%, 60%, 70%, 80%, and 90% 1RM), and five two-point tests (20%&90%, 20%&80%, 20%&70%, 20%&60%, and 20%&40% 1RM). The mean velocity (MV) of each submaximal load was collected to model the individual L-V relationship.

**Results:**

The concurrent validity of the modified multiple-point method worked in incremental loads test until 70% and 80%1RM and for the in-field two-point method it only worked in 20%&90% 1RM conditions (−0.45 ≤ effect size ≤ 0.59; r ≥ 0.810). MV of heavy load in the in-field two-point method was higher than the standard method.

**Conclusion:**

Modified multiple-point method assessing squat L-V relationship variables can choose the incremental load from 20% 1RM to 70%1RM (light load velocity at around 1.0 m/s and heavy load MV around 0.6 m/s) but in-filed two-point method should choose the lightest and heaviest load, around 20%1RM and 90%1RM (light load MV around 1.0 m/s and heavy load MV around 0.4 m/s).

## Introduction

Velocity-based training (VBT) is a modern approach to resistance training that allows for precise and objective determination of training loads and volumes through barbell velocity [1]. Compared to percentage-based training, VBT lets strength and conditioning coaches use movement velocity to guide resistance training, making it easier to monitor fatigue and target specific training adaptations [2,3]. A commonly used model in VBT is the load-velocity (L-V) relationship, which describes the near-linear decline in lifting velocity as external load increases, progressing until the one-repetition maximum (1RM) is reached [4]. By deriving key variables from the L-V relationship, such as the load-axis intercept (*L*_*0*_), velocity-axis intercept (*v*_*0*_), the area under the L-V curve (*A*_line_ = *L*_*0*_ × *v*_*0*_/2), and slope of L-V relationship (*S*). we can effectively describe muscle function in terms of maximum force generation capacity (*L*_*0*_), velocity output (*v*_*0*_), and power production (*A*_line_) [5]. Additionally, L-V relationships are broadly used to regulate training intensity, quantify training-induced fatigue, and assess changes in neuromuscular performance after training interventions [6–8]. However, current literature provides limited information regarding the concurrent validity of L-V relationship variables in squat movements.

Traditionally, assessing the L-V relationship requires measuring incremental load velocity until a heavy load [9]. 90% 1RM is widely accepted as the standard heaviest load [1]. However, due to the risk of injury or poor technique, such heavy loads may not be appropriate or safe for certain populations, including novices and athletes undergoing rehabilitation. Additionally, increasing testing points and load may compromise sports performance in acute fatigue. Miras-Moreno et al., observed lower submaximal barbell velocity in the L-V relationship following the execution of bench pulls at 40% and 90% of 1RM [10]. A lighter heaviest load may be a more suitable option for assessing an athlete’s daily readiness and performance fluctuations. For example, some studies utilized 80% 1RM and 70% 1RM as the maximum loads when evaluating muscle function through the L-V relationship [8,11,12]. However, no studies have compared the differences and accuracy of these methods. It would be beneficial to identify a lighter load that validates the L-V relationship instead of relying on 90% of 1RM.

The standard procedure for assessing the L-V relationship usually involves testing the velocity across four to nine submaximal loads (e.g., 20%, 40%, 60%,70%, 80%, and 90% of 1RM) [10,13]. However, this may result in extended testing durations or accumulation of fatigue. García-Ramos et al., reported lower countermovement jump height after six-load conditions test in vertical jump [14]. The in-field two-point method, which involves testing only the lowest and heaviest loads, has been proposed to optimize testing procedures and minimize fatigue [15]. Researchers have found that excluding intermediate loads does not significantly affect the accuracy of the L-V relationships [13]. For example, Pérez-Castilla et al., found that the L-V relationship variables for countermovement jumps calculated using the four-point method were comparable to those obtained using only the two most extreme loads with loaded jumps or squats as the heaviest point [2]. This finding supported the application of the two-point method in field settings. The in-field two-point method has been shown to be valid for F-V tasks in jumping and leg cycle ergometer, and for the L-V relationship in bench pulling [10,15,16]. Recent studies examining the squat L–V relationship using the two-point method across loads ranging from 40% to 90% of 1RM have provided comparable results [17,18]; however, it remains unclear whether the two-point method with a lighter load range could also yield a valid L–V relationship.

To address the discussed issues, we assessed the L-V relationship variables (*L*_*0*_, *v*_*0*_, *A*_*line*_, and *S*) using modified multi-point methods at different highest submaximal loads and in-field two-point methods at different heaviest loads, and compared them to the standard method. The main objective of the present study was to identify the lowest submaximal load and the corresponding mean barbell velocity to validate the aforementioned L-V relationship variables. We hypothesized that selecting a relatively lighter heavy-load point instead of 90%1RM might be sufficient to establish a valid L-V relationship in both the multiple-point and two-point methods.

## Methods

### Subjects

Twenty college amateur athletes volunteered to participate in this study (age: 22.8 ± 1.7 years, height: 1.77 ± 0.06 m, body mass: 78.7 ± 9.9 kg, resistance training experience: 4.6 ± 2.4 years, absolute squat 1RM = 141.1 ± 16.0 kg, relative squat 1RM = 1.79 ± 0.30). Subjects were instructed to avoid any strenuous low-limb exercises during the testing sessions. They were informed of the study procedures and signed a written informed consent form prior to initiating the study (The study started from January 2024 until March). The study protocol adhered to the tenets of the Declaration of Helsinki.

### Study design

A repeated design was employed to investigate the lightest load that validated L-V relationship variables of back squat in modified multiple-point methods at different highest submaximal loads and in-field two-point methods at different heavy loads. Participants attended the laboratory on eight occasions over three consecutive weeks, with all sessions being separated by 48–72 hours. The first session was dedicated to familiarizing the participants with the back squat. The second session aimed to determine their 1RM. The third session was used for modelling the L-V relationship through the standard method (20%, 40%, 60%, 70%, 80% and 90% 1RM) and 4 modified multiple-point methods at different highest submaximal loads (using the same incremental loads but with the heaviest load for modelling at 80, 70, 60, 40% 1RM). Sessions 4–8 were applied to modelled five in-field two-point methods at different heavy loads (20%&90%, 20%&80%, 20%&70%, 20%&60%, and 20%&40% 1RM, respectively). Testing sessions were performed at the same time of the day for each participant (±1 h) and under similar environmental conditions (~26°C and ~70% humidity).

### Familiarity test (Session 1)

Height (Yunpeng Technology Development Co., Ltd, Dalian, China) and body mass (In Body 270, Biospace, California, USA) were assessed at the beginning of the session, then they were instructed on how to properly perform the squat and carried out some practice sets with light and medium loads. For the squat exercise, participants initially stood upright with their hips and knees locked, and the barbell rested across their upper back/shoulders for all the sessions. Upon the investigator’s command of “squat”, participants descended until the middle of their thighs reached parallel to the ground. Subsequently, participants were encouraged to return immediately to the starting position with maximum movement velocity (utilizing the stretch-shortening cycle). A non-elastic cardboard marker was placed at the lowest point of the squat to define the range of motion. Participants were instructed to keep their heels in contact with the ground and not to wear weight belts during any attempt to prevent fluctuations in movement velocity.

### 1RM Test (Session 2)

After 48 hours of the familiarity test, we conducted the 1RM test for the back squat. Participants began with a standardized warm-up, consisting of 3 minutes of running at a pace of 6 km/h on a treadmill (ShuHua Sports, Quanzhou, China), three sets of the World’s Greatest Stretch, three sets of side lunges (10 repetitions per set), and 10 repetitions of empty barbell squats (20 kg, ShuHua Sports, Quanzhou, China). Subsequently, participants completed an incremental load test, comprising five attempts at 50% of their self-reported 1RM, three attempts at 70% of their self-reported 1RM, and one attempt at 90% of their self-reported 1RM. This was followed by lifts at progressively heavier loads ranging from 0.5 to 5 kg until they achieved their 1RM. Rest periods of approximately 3–5 minutes were provided between sets, and participants were given the opportunity to retry if they failed.

### Standard and modified multi-point L-V relationship Test (Session 3)

The multi-point test was conducted 72 hours after the 1RM test. Participants began with a standardized warm-up, consistent with the 1RM test. Subsequently, they performed squats at 20%, 40%, 60%, 70%, 80%, and 90% of their 1RM in sequence (3 repetitions for 20% and 40%1RM, 2 repetitions for 60% and 70%1RM, and one repetition for 80% and 90%1RM). The fastest MV that met the specified movement requirements was recorded for each load. 3–5 min of rest was provided between loads.

### In field two-point test (Session 4–8)

The two-point tests were conducted in repeated orders: 20&90%1RM, 20&80%1RM, 20&70%1RM, 20&60%1RM and 20&40%1RM. The first two-point test was administered 72 hours after session 3, with participants completing a standardized warm-up consistent with the 1RM test. Subsequently, they performed the two specified test loads. Approximately 3 minutes of rest was provided between the two loads. The repetitions for each load and the data collection strategy were consistent with the multi-point test. Each two-point test was conducted with a 48-hour interval.

### Velocity measurement and data-analysis

MV measurements were obtained using a GymAware linear positional transducer (GymAware PowerTool, Kinetic Performance Technology) connected to a Ninth Generation Apple iPad (Apple Inc., California, USA). MV was calculated as the mean velocity from the beginning of the concentric phase until the load reached its maximum height. The GymAware linear positional transducer was positioned on the ground to the right of the participants’ feet, with the Velcro strap attached 50 cm to the right of the barbell center. Position data were time-stamped at a high-resolution time interval of 35 ms and down-sampled to a frequency of 50 Hz, which represents the standard sampling method employed by GymAware equipment.

**Fig 1:**
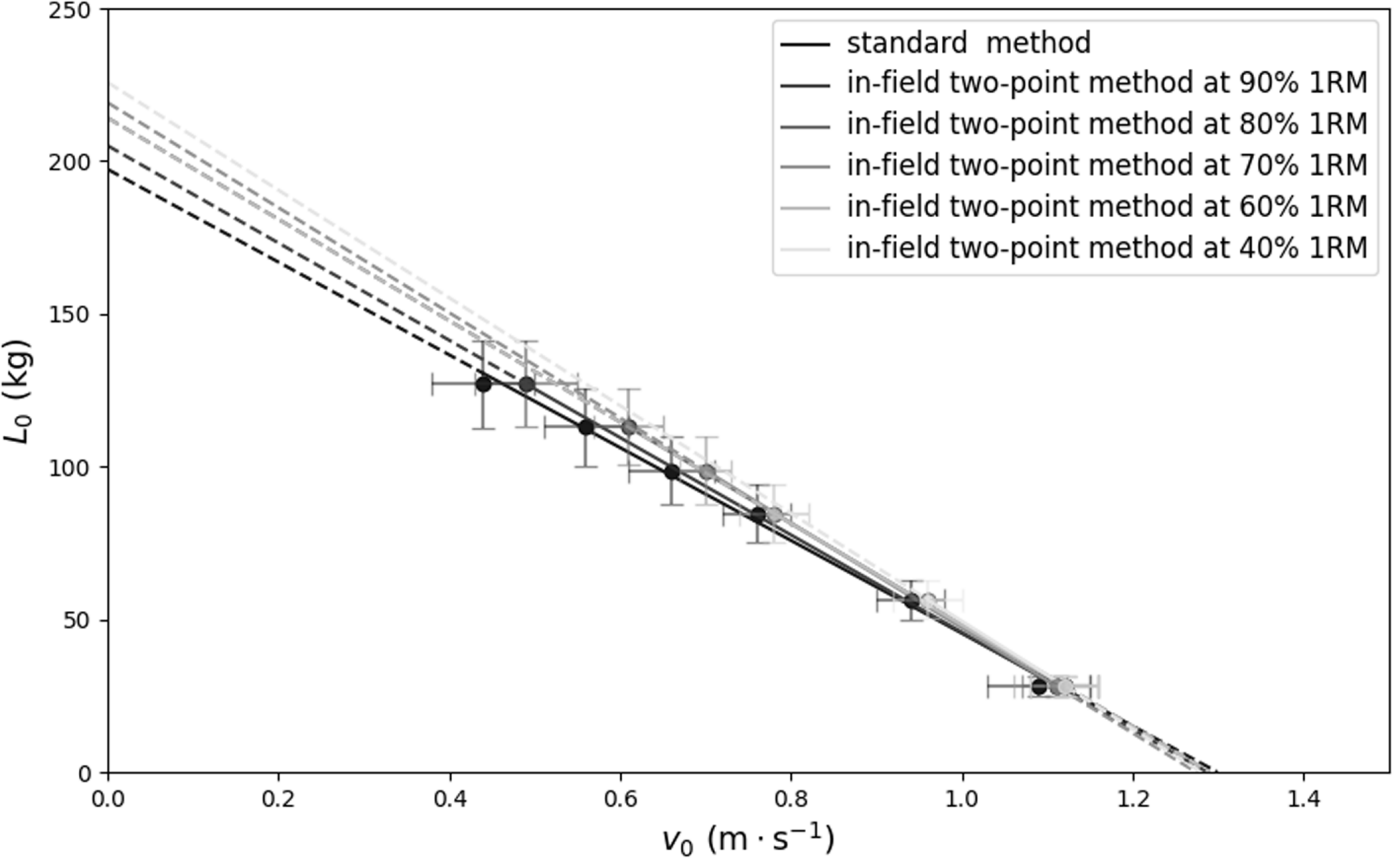
The load-velocity relationships obtained from the data averaged across the participants modelled by the standard method (balck regression line) and modified multiple-point methods at different highest submaixmal load (gray regression lines) during back squat exercise. Mean values are shown for six loading conditions (20, 40, 60, 70, 80, and 90% 1RM) and the error bars represent the standard deviation.

**Fig 2:**
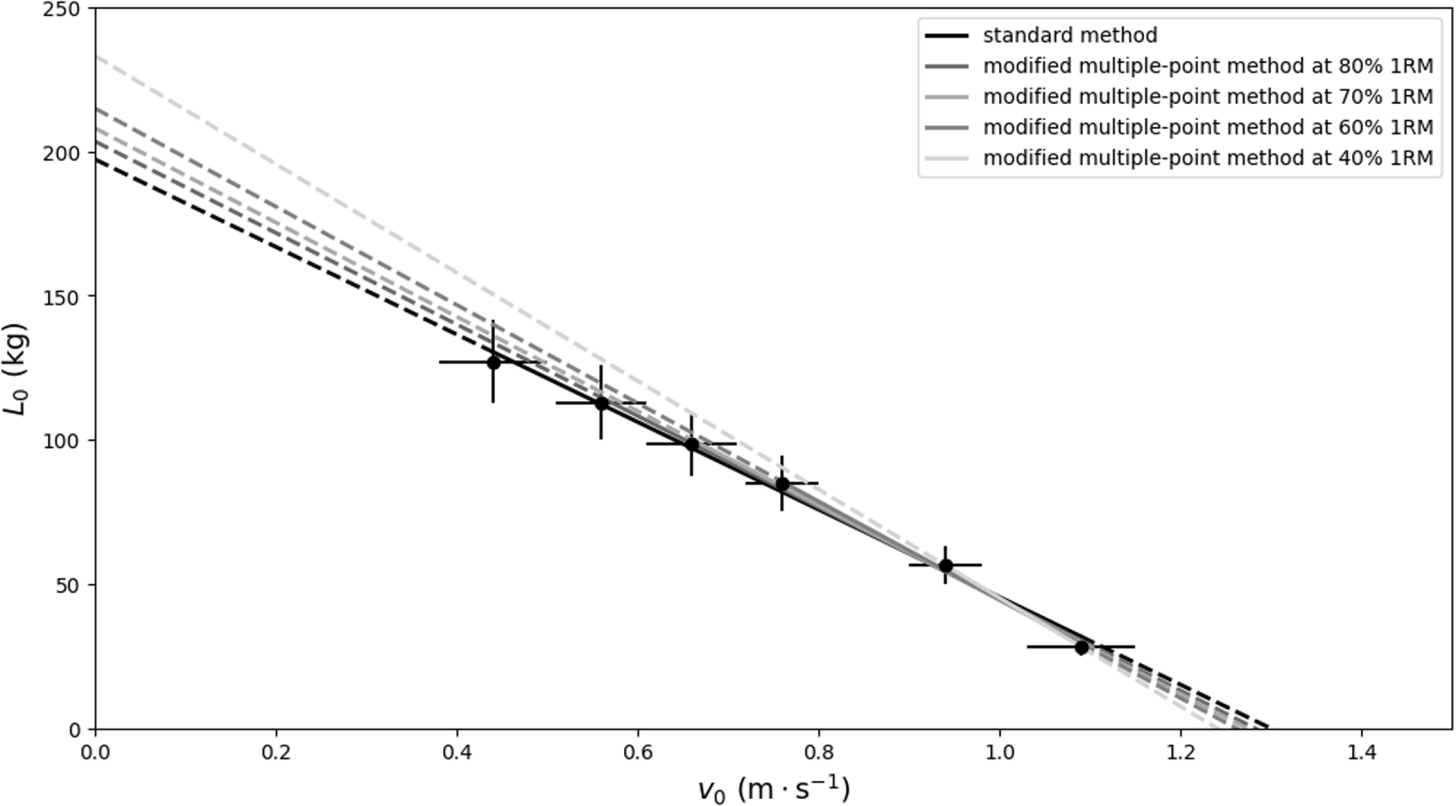
The load-velocity relationships obtained from the data averaged across the participants modelled by the standard method (balck regression line) and five in-field two-point methods at different heavy load (gray regression lines) during back squat exercise. Mean values are shown for six loading conditions (20, 40, 60, 70, 80, and 90% 1RM) and the error bars represent the standard deviation.

A least-squares linear regression model, L(*v*) = *L*_*0*_ – *S *× *v*, was applied to establish individualized load-velocity (L-V) relationships, where *L*_*0*_ represents the theoretical load at zero velocity, and *S* is the slope of the L-V relationship. The values for *v*_*0*_ represent the theoretical velocity at no external load, and *A*_line_ was calculated as *L*_*0 *_× *v*_*0 *_× 0.5. For modeling the L-V relationships, only the repetition with the highest velocity at each load was used. Consequently, one standard method, four modified multiple-point methods at different highest submaximal loads (Fig 1), and five two-point methods (Fig 2) were evaluated for each subject. The modified multiple-point method used the same data from the standard method but the heaviest load and points of load were different. For example, for the modified multiple-point method at 70% 1RM, only 20%, 40%, 60% and 70% 1RM were applied to model the L-V relationship.

### Statistical analysis

Descriptive data are presented as means and standard deviations (SD). The normal distribution of the variables was confirmed by the Shapiro–Wilk test (*P *> 0.05). The strength of load-velocity (L-V) profiles modelled by the standard method (incremental load test until 90%1RM) and three modified multiple-point methods (incremental load test until 60%, 70% and 80%1RM) were examined through the coefficient of determination (r²). The concurrent validity of four modified multiple-point methods (incremental load test until 40%, 60%, 70% and 80%1RM) and five in-field two-point methods (heavy load at 40%, 60%, 70%, 80% and 90%1RM) were examined by Cohen’s d effect size (ES), and correlation coefficients (r) compared to the standard method. The agreement of the standard multiple-point method with respect to the modified multiple-point and in-field 2-point methods was also quantified using the Bland-Altman 95% limits of agreement (LoA) technique (bias ± 1.96 × SD). The acceptable validity of modified multiple-point methods and in-field two-point methods was evaluated using criteria ranging from trivial to small effect sizes (less than 0.60) and from very large to perfect correlation coefficients (greater than 0.70) [10]. Eight one-way repeated measures ANOVA with Bonferroni post hoc corrections were applied to compare whether there was a significant difference between L-V relationship variables (*L*_*0*_, *v*_*0*_, *A*_*line*_, and *S*) between the standard method and modified multiple-point method and two-point method separately. Additionally, paired samples t-tests with ES were used to assess differences in MV across loads from 40% to 90% 1RM in both the standard and in-field two-point methods. Between-session variability was applied to compare through the within-subject Coefficient of Variation (CV) for each load. The criteria to interpret the magnitude of the ES were trivial (<0.20), small (0.20–0.59), moderate (0.60–1.19), large (1.20–2.00), or very large (>2.00). The strength of the r coefficients was interpreted as trivial (0.00–0.09), small (0.10–0.29), moderate (0.30–0.49), large (0.50–0.69), very large (0.70–0.89), nearly perfect (0.90–0.99), and perfect (1.00). Acceptable reliability was considered as CV ≤ 10% while good reliability was considered as CV ≤ 5% [19]. All statistical analyses were performed using SPSS software (IBM SPSS version 22.0, Chicago, IL). Alpha was set at 0.05.

## Results

The strength of the individualized L-V relationship was nearly perfect for all modified multiple-point methods (r^2^ range from 0.942 to 1.000) and the standard method (r^2^ range = 0.961 to 0.999). Since two points always determine a straight line, the coefficient of determination is necessarily equal to 1. The L-V relationship variables obtained from different methods were shown in Table 1.

**Table 1:** The load-velocity (L-V) relationship variables obatined from different method (standard method, multiple-point methods at different highest submaixmal load and in-field two-point methods at different heavy load) during back squat exercise.

Variable	*L*_*0*_ (kg)	*v*_*0*_ (m·s^-1^)	*A*_*line*_ (kg·m·s^-1^)	*S* (kg·s·m^-1^)
Standard method	196.88 ± 21.99	1.31 ± 0.06	128.39 ± 12.52	151.33 ± 21.99
Multiple-point method at 80% 1RM	202.95 ± 23.79	1.29 ± 0.06	130.58 ± 13.30	158.08 ± 23.87
Multiple-point method at 70% 1RM	208.59 ± 25.44	1.28 ± 0.07	132.87 ± 13.26	164.21 ± 26.94
Multiple-point method at 60% 1RM	216.21 ± 31.16	1.27 ± 0.07	136.31 ± 15.37	172.05 ± 33.22
Multiple-point method at 40% 1RM	238.82 ± 58.02	1.25 ± 0.09	147.30 ± 26.81	194.68 ± 61.14
Two-point method at 90% 1RM	206.38 ± 23.22	1.28 ± 0.06	132.46 ± 13.95	160.77 ± 21.98
Two-point method at 80% 1RM	213.88 ± 27.31	1.30 ± 0.06	138.68 ± 15.53	164.92 ± 25.63
Two-point method at 70% 1RM	220.92 ± 27.78	1.28 ± 0.07	141.03 ± 15.27	173.02 ± 27.84
Two-point method at 60% 1RM	217.89 ± 30.73	1.29 ± 0.06	140.98 ± 17.54	168.39 ± 28.76
Two-point method at 40% 1RM	232.67 ± 44.84	1.28 ± 0.07	148.64 ± 23.65	182.11 ± 42.64

*L*_*0*_ = load-axis intercept; *v*_*0*_ = velocity-axis intercept; *A*_*line*_ = area under the L-V relationship line. *S *= slope of load-velocity profile.

All L-V relationship variables (*L*_*0*_, *v*_*0*_, *A*_*line*_, and *S*) obtained from the modified multiple-point method at 70% and 80% 1RM showed nearly perfect correlation (r = 0.911 to 0.983) with the standard method and from trivial to small change (ES = −0.45 to 0.59). However, the modified multiple-point method at 40% (r = 0.370 to 0.719 and ES = −0.81 to 1.97) and 60% (r = 0.748 to 0.838 and ES = −0.62 to 0.94) did not meet the criteria (Table 2). ANOVA indicated significant systematic errors compared with the standard method for all variables and all modified multiple-point methods (F ≥ 8.822, p ≤ 0.001).

**Table 2:** Comparison of the load-velocity (L-V) relationship variables obtained by the modified multiple-point methods at different highest submaixmal load with respect to the standard method during back squat exercise.

Method	Variable	ES	r (95% CI)	Bias (95% LoA)
80%1RM	*L*_*0*_	0.28	0.961 (0.906, 0.986)	6.07 (−6.92, 19.06)
	*v*_*0*_	−0.27	0.958 (0.917, 0.982)	−0.02 (−0.05, 0.02)
	*A*_*line*_	0.17	0.983 (0.954, 0.994)	2.19 (−2.70,7.08)
	*S*	0.31	0.952 (0.880, 0.983)	6.75 (−7.60,21.10)
70%1RM	*L*_*0*_	0.53	0.911 (0.813, 0.963)	11.17 (−8.98, 32.39)
	*v*_*0*_	−0.45	0.932 (0.853, 0.971)	−0.03 (−0.08, 0.02)
	*A*_*line*_	0.36	0.950 (0.874, 0.979)	4.48 (−3.60, 12.53)
	*S*	0.59	0.906 (0.801, 0.962)	12.88 (−10.01, 35.77)
60%1RM	*L*_*0*_	0.88	0.748 (0.518, 0.892)	19.32 (−21.28, 59.93)
	*v*_*0*_	−0.62	0.838 (0.652, 0.931)	−0.04 (−0.12, 0.04)
	*A*_*line*_	0.63	0.826 (0.655, 0.936)	7.92 (−9.07, 24.91)
	*S*	0.94	0.748 (0.494, 0.962)	20.72 (−22.87,64.30)
40%1RM	*L*_*0*_	1.91	0.399 (0.032, 0.785)	41.94 (−62.38, 146.26)
	*v*_*0*_	−0.87	0.719 (0.437, 0.908)	−0.06 (−0.18, 0.07)
	*A*_*line*_	1.51	0.370 (−0.101, 0.744)	18.91 (−30.17, 67.99)
	*S*	1.97	0.481 (0.147, 0.830)	43.35 (−62.72,149.42)

ES = Cohen d effect size; r = Pearson product-moment correlation coefficients; 95% Cl = 95% confidence interval; 95% LoA = 95% limits of agreement (±1.96 × SD); *L*_*0*_ = load-axis intercept; *v*_*0*_ = velocity-axis intercept; *A*_*line*_ = area under the L-V relationship line. *S *= slope of load-velocity profile.

All L-V relationship variables obtained from the two-point method at 90% 1RM showed very large correlations (r = 0.810 to 0.866) with the standard method and small change (ES = −0.37 to 0.46) compared with the standard method. Only the *v*_*0*_ with the two-point method at 70% and 80% 1RM showed very large correlations (r = 0.775 to 0.816) and small change (ES = −0.47 to −0.16). The remaining variables showed moderate to very large correlation (r = 0.227 to 0.872) and small to large change (ES = −0.46 to 1.63), which did not meet the criteria (Table 3). ANOVA indicated significant systematic errors compared with the standard method for all variables and all two-point methods (F ≥ 2.309, p ≤ 0.050).

**Table 3:** Comparison of the load-velocity (L-V) relationship variables obtained by in-field two-point method at different heavy load with respect to the standard method during back squat exercise.

Method	Variable	ES	r (95% CI)	Bias (95% LoA)
90%1RM	*L*_*0*_	0.43	0.866 (0.695, 0.953)	9.50 (−13.60, 32.59)
	*v*_*0*_	−0.37	0.810 (0.701, 0.920)	−0.02 (−0.10, 0.05)
	*A*_*line*_	0.30	0.860 (0.673, 0.954)	3.85 (−10.13, 17.83)
	*S*	0.46	0.848 (0.706, 0.930)	10.02 (−13.72, 33.77)
80%1RM	*L*_*0*_	0.77	0.870 (0.685, 0.959)	17.00 (−9.63, 43.62)
	*v*_*0*_	−0.16	0.816 (0.646, 0.913)	−0.01 (−0.09, 0.06)
	*A*_*line*_	0.80	0.839 (0.691, 0.942)	9.93 (−6.67, 26.53)
	*S*	0.65	0.873 (0.658, 0.970)	14.36 (−10.19, 38.91)
70%1RM	*L*_*0*_	1.09	0.869 (0.714, 0.952)	24.03 (−3.27, 51.34)
	*v*_*0*_	−0.47	0.775 (0.607, 0.901)	−0.03 (−0.12, 0.06)
	*A*_*line*_	0.98	0.858 (0.700, 0.954)	12.23 (−3.16, 27.63)
	*S*	1.02	0.853 (0.635, 0.949)	22.65 (−6.04, 5134)
60%1RM	*L*_*0*_	0.96	0.634 (0.321, 0.832)	21.01 (−25.83, 67.85)
	*v*_*0*_	−0.21	0.481 (0.189, 0.741)	−0.01 (−0.13, 0.11)
	*A*_*line*_	0.98	0.756 (0.495, 0.894)	12.21 (−10.34, 34.75)
	*S*	0.81	0.526 (0.163, 0.754)	17.84 (−31.97, 67.64)
40%1RM	*L*_*0*_	1.63	0.340 (−0.181,0.714)	35.79 (−47.90, 119.49)
	*v*_*0*_	−0.46	0.407 (−0.007, 0.954)	−0.03 (−0.17, 0.11)
	*A*_*line*_	1.59	0.480 (−0.014, 0.930)	19.28 (−21.46,60.02)
	*S*	1.43	0.277 (−0.210, 0.701)	32.43 (−50.33, 115.19)

ES = Cohen d effect size; r = Pearson product-moment correlation coefficients; 95% Cl = 95% confidence interval; 95% LoA = 95% limits of agreement (±1.96 × SD); *L*_*0*_ = load-axis intercept; *v*_*0*_ = velocity-axis intercept; *A*_*line*_ = area under the L-V relationship line. *S *= slope of load-velocity profile.

The MV for the same load was found to be significantly higher for the in-field two-point method compared to the multi-point method test (p < 0.038). There was a small to moderate effect size of heavy load in the in-field two-point method L-V relationship compared to the multi-point method L-V relationship (ES = 0.5 to 1.2). The MV of all loads including 20% 1RM (CV = 2.03%) showed good reliability (CV ≤ 4.14%) (Table 4).

**Table 4:** Barbell velocity of heavy load from in-field two-point method and standard method.

Variable	40%1RM	60%1RM	70%1RM	80%1RM	90%1RM
In-field two-point method (m·s^-1^)	0.96 ± 0.04	0.78 ± 0.04	0.70 ± 0.03	0.61 ± 0.04	0.49 ± 0.06
multiple point method (m·s^-1^)	0.93 ± 0.04	0.76 ± 0.04	0.66 ± 0.05	0.56 ± 0.05	0.44 ± 0.06
t	2.613	2.231	5.257	4.628	4.605
p	0.017	0.038	<0.001	<0.001	<0.001
ES	0.75	0.5	0.8	1.2	0.83
CV (%)	2.02	2.37	2.97	3.85	4.14

Abbreviations: ES, effect size; CV, Coefficient of variation.

## Discussion

This study aimed to optimize the test procedure for measuring the L-V relationship and found the lightest barbell load that validated squat L-V relationship variables for the modified multiple-point method and in-field two-point method. Our results showed that the modified multiple-point method for assessing squat L-V relationship variables could choose the incremental load from 20% 1RM to 70% 1RM with four points (light load velocity at around 1.0 m/s and heavy load MV around 0.6 m/s) or use 20%1RM and 90% 1RM for the in-field two-point method (light load MV around 1.0 m/s and heavy load MV around 0.45 m/s). Also, the standard method reduced the barbell velocity at submaximal loads compared with the in-field two-point method.

Compared with the standard method for assessing the L-V relationship up to 90% 1RM [8,20,21], our results demonstrated an innovative approach in back squat testing, where the L-V relationship can be accurately measured using velocities corresponding to loads from 20% 1RM (approximately 1.0 m/s) to 70% 1RM (approximately 0.6 m/s). Although 90% 1RM is the standard method for assessing the L-V relationship, many experiments preferred to use 80% 1RM as the final load [4,8,11,22,23]. In fact, some studies even applied the incremental loading at 70% 1RM [12,24]. For example, Sayers et al., demonstrated that measuring bench press throws velocity at 30% and 70% of 1RM is adequate for accurately predicting 1RM in the bench press [12]. However, to the best of the author’s knowledge, most studies did not assess the validity of their methods in evaluating the L-V relationship. The most closely related research has demonstrated that 1RM prediction using load at zero velocity was comparable for the six-point method (ranging from 40% to 90% 1RM) and the four-point method (ranging from 50% to 80% 1RM) in bench press F-V relationship [11]. However, they did not compare L-V variables directly. Our results provided strong evidence for sports scientists and coaches to measure lower limb muscle function using a modified multiple-point method with less fatigue.

Consistent with existing in-field two-point methods [10,15], our results demonstrated that the in-field two-point method is effective for assessing the load-velocity L-V relationship during the back squat. Beyond this, we also found that L-V relationship variables assessed with a lower heavy load (e.g., 80% 1RM) using the in-field two-point method revealed a moderate to large increase compared to those assessed using the standard method up to 90% 1RM except *v*_*0*_. Only the lightest and heaviest loads in the two-point method could accurately assess the L-V relationship of the back squat. García-Ramos et al., found similar results in the jump F-V relationship, where only the heaviest load (75 kg) was effective for assessing F-V variables using the two-point method [25]. It is pointed out that the distance between the two loads used was crucial for ensuring valid testing outcomes [13]. Our experiments proved this theory in squat, the velocity in this method should range from 0.45 m/s to 1.0 m/s for the in-field two-point method.

Another noteworthy finding from our research is that the submaximal load velocity was higher for the in-field two-point method compared to the standard method. This result aligns with the findings of [9,10]. Our research also found a small to large increase in the heavy barbell velocity for the in-field two-point method. For example, the 90% 1RM showed a 10% increase in the in-field two-point method compared to the standard multiple-point method. 90%1RM and other heavy loads are the closest point to *L*_*0*_. Errors in these measurements determined the extent of the *L*_*0*_ offset. This led to a large overestimate of *L*_*0*_ of the in-field two-point method compared to the standard method. The two-point method may provide a more accurate reflection of muscle function compared to the standard multiple-point method because it involves less fatigue accumulation and provides a simpler, more efficient measurement process [9]. However, it is important to note that the barbell velocity at 40% 1RM increased by approximately 3% with the in-field two-point method compared to the standard method (p = 0.017), as it followed the lightest load (20 kg), similar to the procedure in the standard method. Given that both methods likely induced similar levels of fatigue, it remains unclear whether the increase in velocity was due to greater familiarity with the movement or reduced fatigue.

When interpreting the findings of this study, several limitations should be considered. Firstly, our subjects were college amateur athletes, potentially differing in RT experience and strength levels from professional athletes. Secondly, our research only examined specific load combinations, including 20% 1RM in combination with other loads (40%, 60%, 70%, 80%, or 90% 1RM), rather than exploring all possible load combinations. 40% of 1RM is commonly utilized as an initial load for predicting 1RM [1]. Lastly, despite this comparable validity, a significant degree of bias has been observed. This bias may, in certain individual cases, lead to the misrepresentation of inaccurate 1RM estimation [26], as these kinds of methods can be highly sensitive to subtle errors and external disturbances.

## Conclusion

In evaluating lower limb muscle function through the L-V relationship, incremental tests using 20% to 70% (around 0.7 m/s) or 80% 1RM (around 0.6m/s) provide validity comparable to using 90% 1RM. Additionally, the in-field two-point method with 20% and 90% 1RM (around 0.4 m/s) can effectively assess the L-V relationship. Our study simplifies the L-V relationship testing procedure for lower limbs, making it more suitable for field testing and daily training due to its efficiency, simplicity, and minimal fatigue.
